# Supplementary figures and images for: Perineuronal nets in HVC and plasticity in male canary song
Source: PLoS One. 2021 Aug 27;16(8):e0252560. doi: 10.1371/journal.pone.0252560 (PMC8396724; doi:10.1371/journal.pone.0252560)

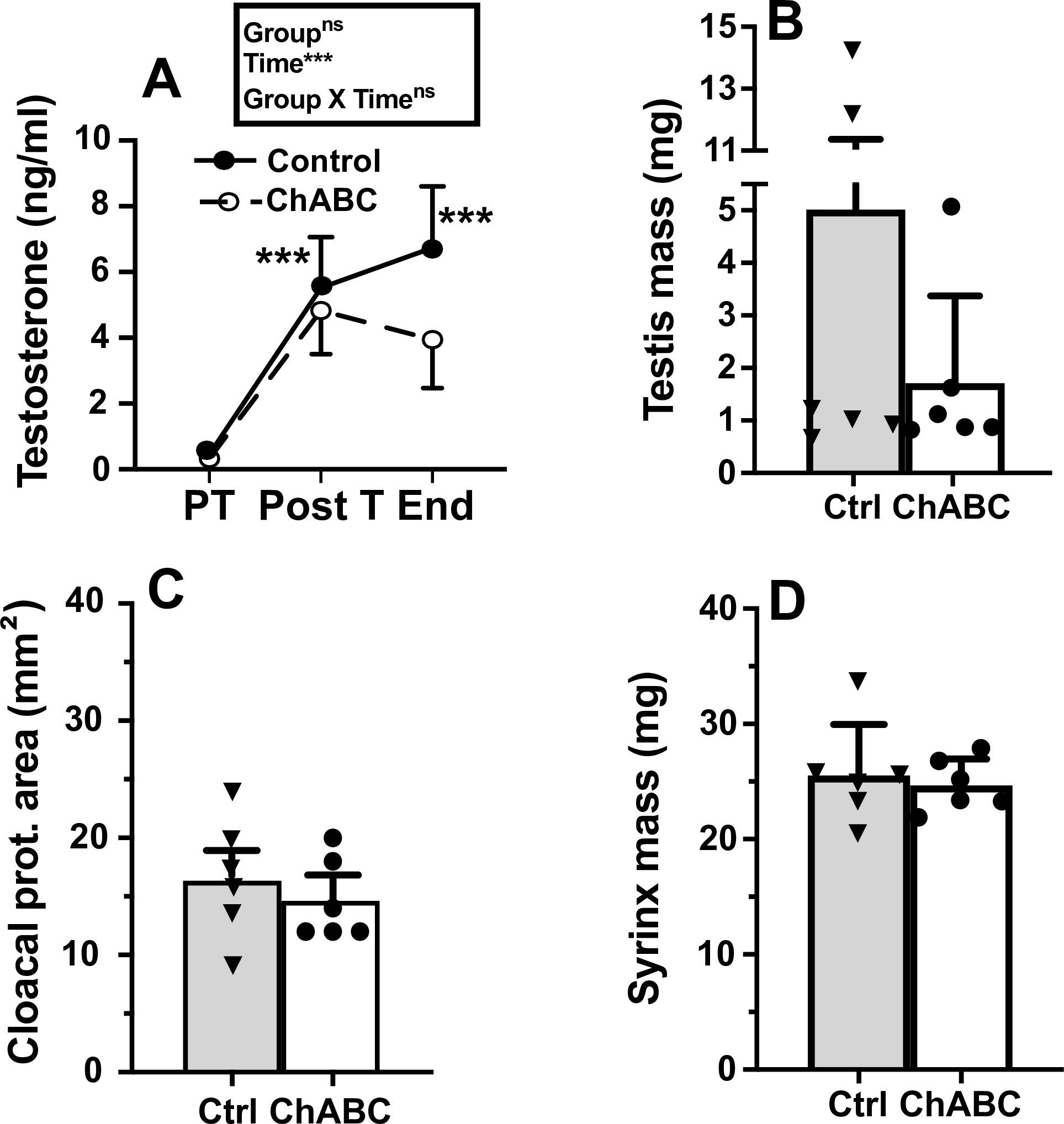

Supplement: S1 Fig — A. Testosterone concentrations before surgery (PT), after T implants (Post T), and at brain collection (End) in Ctrl and ChABC males (A). Results of the two-way ANOVA of these data are indicated in the insert (*** = p<0.001 for the comparisons with the PT time point by post-hoc tests). B. Testis mass, C: Cloacal protuberance area and D: Syrinx mass at brain collection in the Ctrl and ChABC groups. Individual values are presented for the last three measures that were analyzed by t-tests but indicated no significant difference. (TIF) [file pone.0252560.s001.tif]

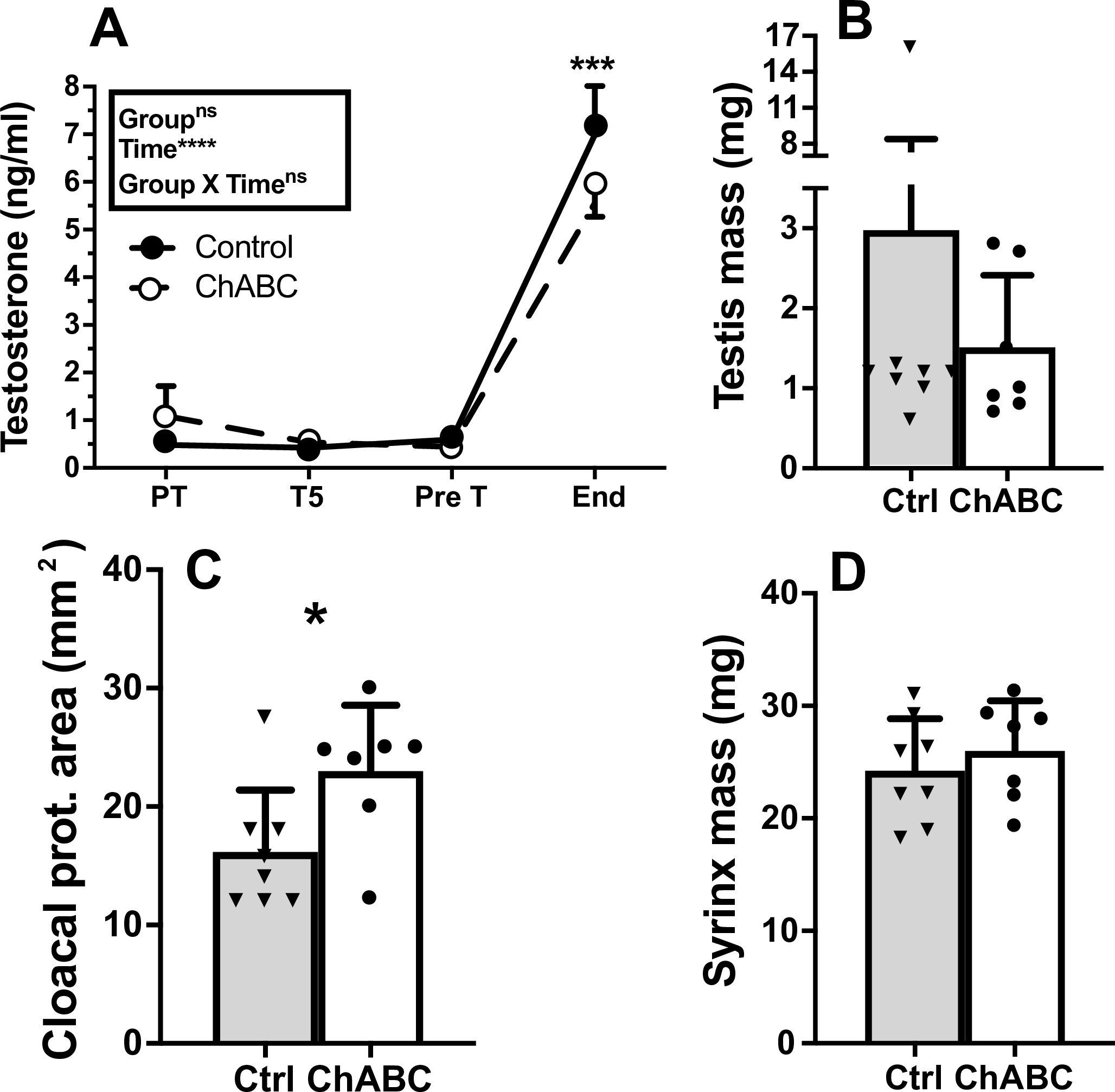

Supplement: S2 Fig — A. Testosterone concentrations before surgery (PT), 5 weeks after surgery (T5), before T implants (Pre T), and at brain collection (End) in Ctrl and ChABC groups (A). Results of the two-way ANOVA of these data are indicated in the insert (*** = p<0.001 for the comparisons with all other time points by post-hoc tests). B. Testis mass, C: Cloacal protuberance area and D: Syrinx mass at brain collection in the Ctrl and ChABC groups. Individual values are presented for the last three measures that were analyzed by T tests (* = p<0.05). (TIF) [file pone.0252560.s002.tif]

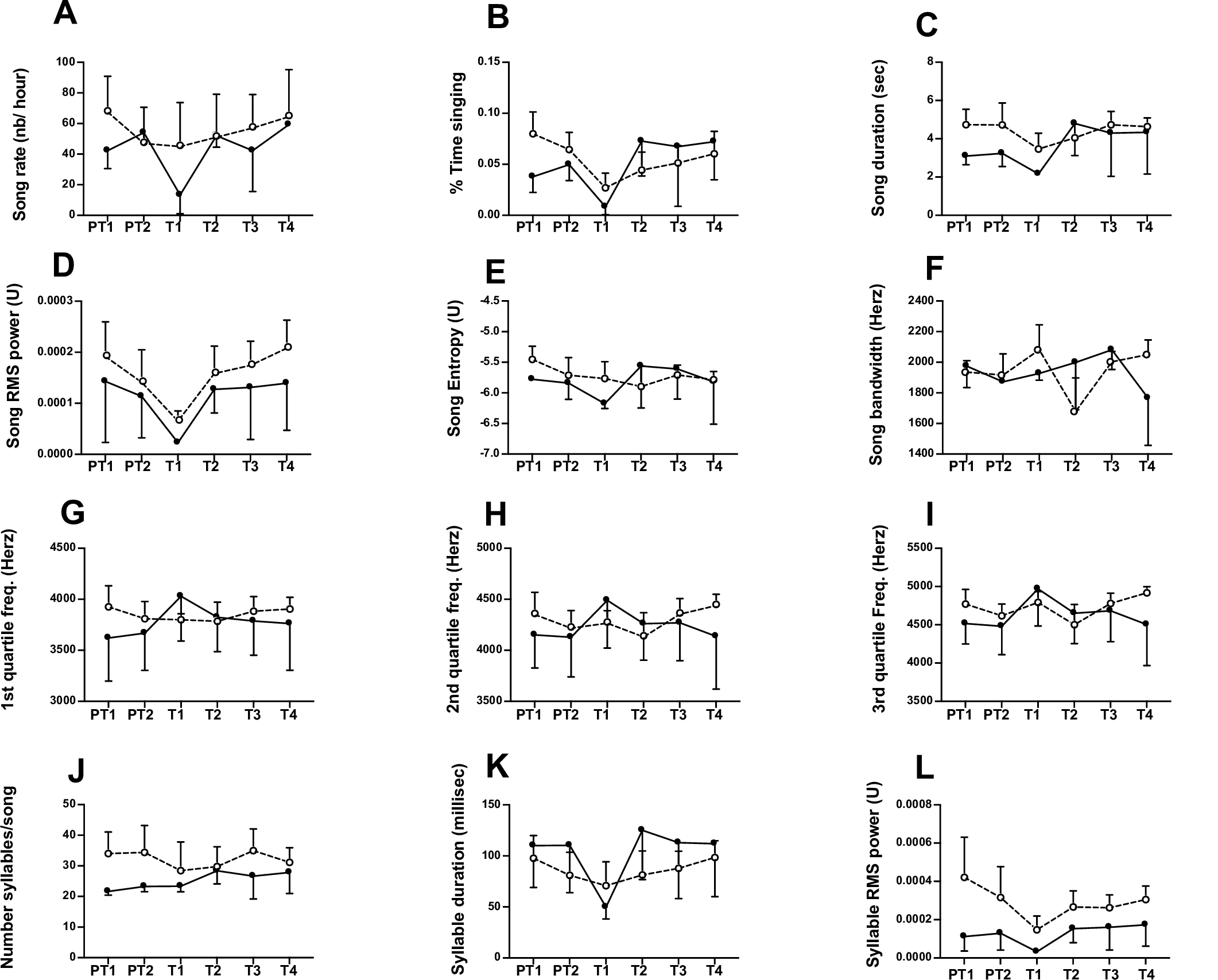

Supplement: S3 Fig — Data were analyzed by two-way ANOVA with groups as independent factor and time as a repeated factor and results are summarized in the insert for each panel. These analyses identified no significant effect of the two main factors and of their interaction (all p>0.05) but have a limited power due to the reduced sample size (2 Ctrl and 7 ChABC males). (TIF) [file pone.0252560.s003.tif]

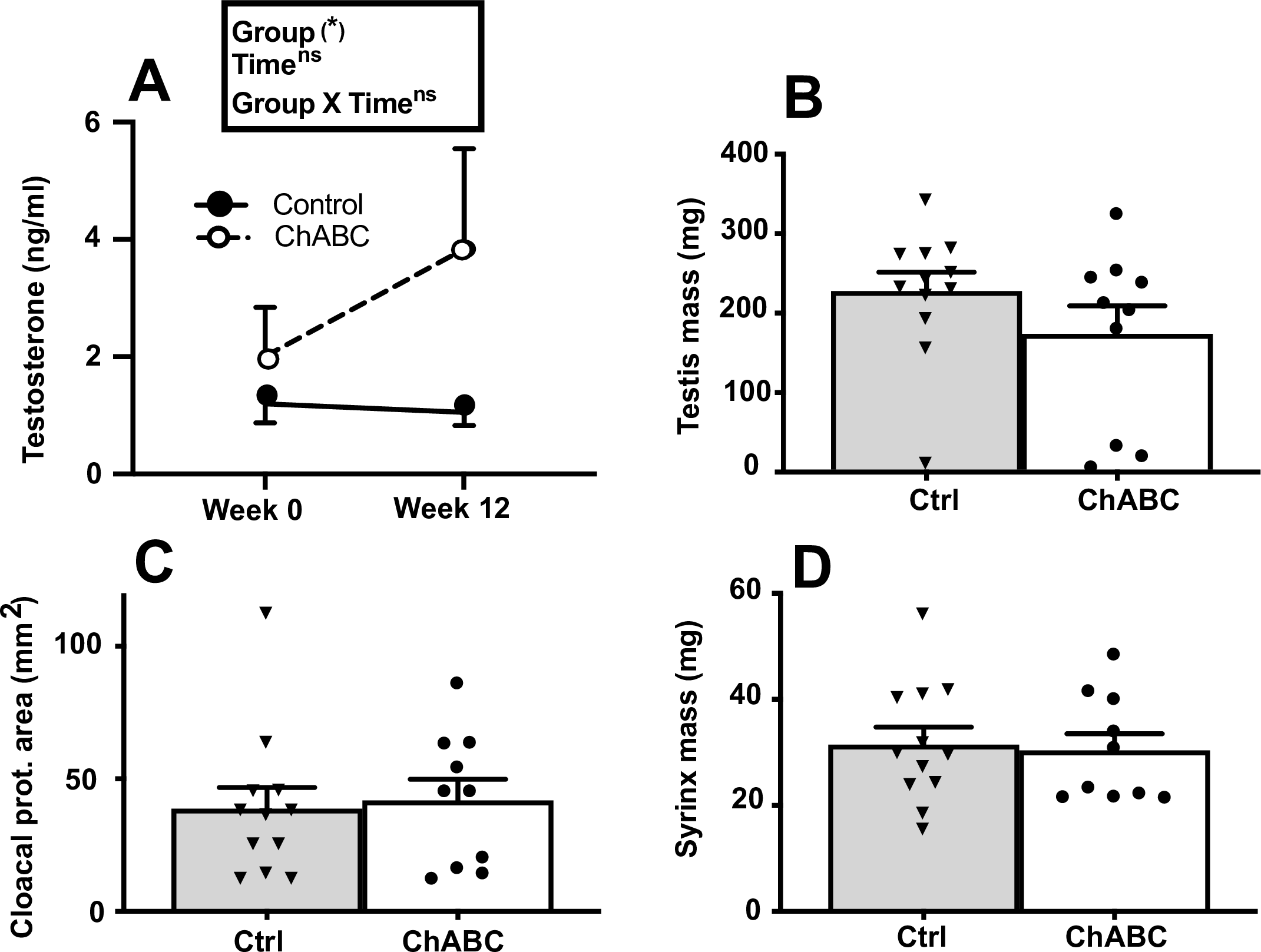

Supplement: S4 Fig — A. Testosterone concentrations at the beginning (Week 0) and the end (Week 12) of the experiment in Ctrl and ChABC groups. Results of the two-way ANOVA of these data are indicated in the insert (see also text for additional detail). B. Testis mass, C. Cloacal protuberance area and D. syrinx mass at brain collection in the Ctrl and ChABC groups. Individual values are presented for the last three measures that were analyzed by T tests but indicated no significant difference. (TIF) [file pone.0252560.s004.tif]
